# Enhanced catalytic activity of MgO-grafted aluminium isopropoxide in heterogeneous H-transfer reduction reactions through surface support modification

**DOI:** 10.1039/d4ra08813a

**Published:** 2025-04-22

**Authors:** Xiao Yu, Atika Muhammad, Boya Qiu, Aristarchos Mavridis, Min Hu, Carmine D'Agostino

**Affiliations:** a Department of Chemical Engineering, The University of Manchester Oxford Road Manchester M13 9PL UK min.hu@manchester.ac.uk carmine.dagostino@manchester.ac.uk; b Dipartimento di Ingegneria Civile, Chimica, Ambientale e dei Materiali (DICAM), Alma Mater Studiorum-Università di Bologna Via Terracini, 28 Bologna 40131 Italy

## Abstract

The heterogenization of aluminium isopropoxide [Al(O^*i*^Pr)_3_] on modified magnesium oxide (MgO) supports was investigated to develop efficient catalysts for hydrogen-transfer (H-transfer) reduction reactions. MgO surfaces were functionalized with octadecyltrichlorosilane (OTES) or dibromobutane (DBB) to optimize the surface chemistry of commercial MgO. The OTES-modified MgO exhibited a porous “nest-like” structure with a markedly increased surface area of 84.3 m^2^ g^−1^, compared to 2.9 m^2^ g^−1^ for the unmodified MgO. In contrast, DBB-modified MgO displayed a “brush-like” morphology attributed to the flexibility of the immobilized carbon chains. The modified heterogenized catalysts demonstrated substantial improvements in activity compared to the unmodified MgO-based systems. Among the heterogenized catalysts, Al–DBB–MgO achieved the highest turnover frequency (TOF), which is attributed to enhanced substrate adsorption and reduced steric hindrance, facilitating efficient reactant access to active sites. The activity of the two modified catalysts after 5 reduction cycles shows no obvious change in terms of the TOF of Al–DBB–MgO, while the TOF of Al–OTES–MgO dropped by around 14%. These findings highlight the critical role of MgO surface modification in enabling effective Al(O^*i*^Pr)_3_ immobilization and enhancing catalytic performance for H-transfer reactions, offering a promising strategy for designing advanced heterogenized catalysts for reduction of carbonyl compounds.

## Abstract 2

The heterogenization of aluminium isopropoxide [Al(O^*i*^Pr)_3_] on modified magnesium oxide (MgO) supports was investigated to develop efficient catalysts for hydrogen-transfer (H-transfer) reduction reactions.

## Graphical abstract

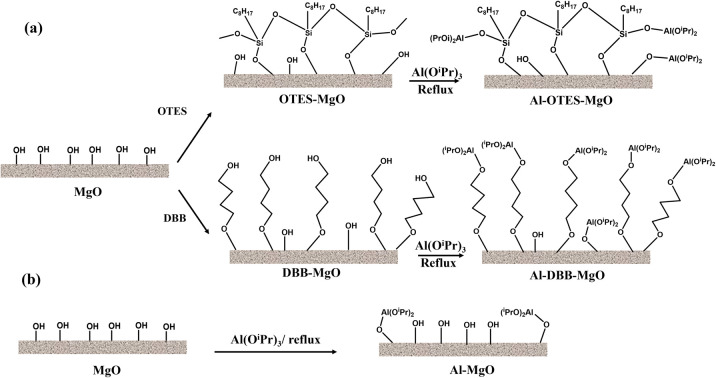

## 1. Introduction

Catalytic transfer hydrogenation has emerged as an attractive alternative for synthesizing alcohols from aldehydes and ketones, offering a safer and more cost-effective approach compared to conventional direct hydrogenation.^1,2^ This process eliminates the need for pressurized hydrogen, instead utilizing secondary alcohols as hydrogen donors. Furthermore, it exhibits high chemoselectivity, particularly in the reduction of unsaturated aldehydes or ketones to produce allylic alcohols-valuable chiral building blocks in organic synthesis.^3,4^ Traditionally, the reaction is catalysed by homogeneous metal alkoxide catalysts, such as aluminium (Al), boron (B), or zirconium (Zr) alkoxides, which demonstrate superior catalytic performance due to their Lewis acidic character in combination with ligand exchangeability.^5,6^ However, these systems often require an excess of alkoxides and neutralization of the residual alkoxide with strong acid, posing practical and environmental challenges.^7^ To address these limitations, recent efforts have focused on developing heterogeneous catalysts, which can be easily separated from the reaction mixture, offering improved reusability and sustainability.^8^

Initial studies on catalyst design for H-transfer reductions predominantly explored metal oxides and zeolites, capitalizing on their intrinsic Lewis acidic properties as an extension of homogeneous catalytic systems. For example, Lavalley *et al.*^9^ systemically investigated heterogeneous acetone reduction over various metal oxide catalysts, demonstrating that the catalytic activity is strongly correlated with surface acid–base properties. Lewis acidic catalysts, such as chloride-modified Al_2_O_3_ (Al_2_O_3_–Cl), and basic catalysts, including MgO and ZrO_2_, exhibited comparable activity. Similarly, H van Bekkumn's group^10^ reported the reduction of substituted cyclohexanones over zeolite BEA, achieving superior seteroselectivity towards *cis*-4-*tert*-butylcyclohexanol. This performance was attributed to Lewis acidic aluminium sites located within the zeolite's micropores. Efforts to enhance zeolite catalytic performance have included incorporating elements like Sn and Zr into the framework to increase Lewis acidity.^11,12^

Heterogenized homogeneous catalysts represent another significant advancement, achieved by covalently grafting metal alkoxide catalysts onto solid supports. Porous silicas are among the most used supports due to their diverse porous structures.^13,14^ Such heterogenized catalysts can often retain the high activity and selectivity of their homogeneous counterparts, particularly in the synthesis of α,β-unsaturated alcohols. For example, Uysal and Oksal,^15^ developed a boron-based heterogeneous catalyst by grafting the boron tri-ethoxide onto mesoporous MCM-41 [B(OEt)_3_-MCM-41], which exhibited comparable catalytic activity to homogeneous B(O^*i*^Pr)_3_ and B(OEt)_3_ catalysts. This system demonstrated excellent stability and recyclability, maintaining catalytic activity over six cycles without significant performance loss. Similarly, in our previous work,^16^ we immobilised aluminium isopropoxide [Al(O^*i*^Pr)_3_] onto various mesoporous supports, including SiO_2_, TiO_2_ and γ-Al_2_O_3_, and evaluated their performance in H-transfer reductions. The heterogenized catalysts exhibited excellent activity and high selectivity, closely mirroring the performance of homogeneous Al(O^*i*^Pr)_3_. Among these, Al(O^*i*^Pr)_3_ grafted onto SiO_2_ outperformed those on γ-Al_2_O_3_ and TiO_2_, owning to its higher surface area and large pore structure, which facilitated improved Al(O^*i*^Pr)_3_ dispersion and enhanced accessibility of the carbonyl substrates to active sites. These findings underscore the importance of carefully selecting and engineering support materials to achieve optimal catalytic performance.

In this study, we explore the impact of MgO surface modification using different functional groups, octyltriethoxysilane, OTES and 1,4-dibromobutane, DBB, to tailor MgO surface chemistry for improved catalytic performance. A comprehensive set of characterization techniques, including FTIR, XRD, SEM, and nitrogen adsorption, were employed to probe the structural and surface chemistry changes induced by the modifications. The catalyst activity of the unmodified and modified supports was evaluated in H-transfer reduction reactions with various substrates including aldehydes and ketones. This work aims to provide insights into the relationship between surface modification and catalytic efficiency, paving the way for the development of advanced heterogenized catalysts with improved performances through relatively simple support modifications.

## 2. Experimental

### 2.1 Materials and chemicals

Aluminium isopropoxide (98%), 2-propanol (anhydrous, 99.5%), 1,3,4,5-tertmethylbenze (99%), hexanal (99%), cinnamaldehyde (99%), benzaldehyde (99%), cyclohexanone (99%), MgO catalyst support, 1,4-dibromobutane (DBB) (98%), octyltriethoxysilane (OTES) (97%), chloroform-d (99.8 atom %D) and NaOH were obtained from Sigma Aldrich, UK. All the chemicals were used without further treatment.

### 2.2 MgO modified with OTES (MgO–OTES)

The salinisation of MgO was performed using a chemical grafting method. A 150 mL round-bottom flask was charged with 1.8 mL of octyltriethoxysilane (OTES) and 30 mL of water first, stirring for 3 h. Then 3.0 g of MgO support was added into the mixture and followed by stirring overnight. The modified MgO was then collected by centrifugation and dried at room temperature for 48 h.

### 2.3 MgO modified with DBB (MgO–DBB)

1.38 g of NaOH and 3.0 g of MgO support were added to 30 mL of cyclohexane and stirred for 2 h to get a homogeneous suspension. Then 0.6 mL of 1,4-dibromobutane (DBB) was added and the mixture was refluxed at 90 °C overnight. After that, 5 mL of water was added to quench the reaction and stirred for 1 h. The product was then collected by centrifugation and dried at room temperature for 48 h.

### 2.4 Heterogenized catalysts synthesis with various supports

The immobilization of Al(O^*i*^Pr)_3_ onto different supports were achieved by a grafting method as previously reported.^16^ In a typical synthesis, 3.0 g homogeneous Al(O^*i*^Pr)_3_ catalyst was first dissolved in 30 mL anhydrous cyclohexane (theorical Al loading: ∼20 wt%). Then 2.0 g modified or un-modified MgO support was added into the above solution. The suspension was heated to 90 °C and refluxed overnight. Then the product was collected by centrifugation and washed with cyclohexane three times before drying. The resulted catalysts named as Al–MgO, Al–OTES–MgO and Al–DBB–MgO respectively.

### 2.5 Characterization

The X-ray diffraction (XRD) patterns of both supports and catalysts were recorded on a Philips X'Pert X-ray diffractometer using Cu K*α*_1_ radiation (*λ* = 1.5406 Å) operating at 40 kV, 40 mA with a scanning rate of 2° min^−1^, a step size of 0.02° s^−1^, and a 2*θ* angle from 20° to 80°. The Al amount of the graft catalyst was measured by inductively coupled plasma-optical emission spectroscopy (ICP-OES) on an Analytic Jena Plasma Quant 9000 Elite. The heterogenized catalysts were dissolved in a strong acid solution (*V*_HNO_3__ : *V*_HCl_ = 3 : 1) in a microwave digest reactor. The resulted solution was diluted and filtered before the measurement. An acid solution containing aluminium was used as a standard reference. The surface area of both supports and catalysts was measured by nitrogen physisorption isotherms at −196 °C on a Micrometrics ASAP 2020 according to the Brunauer–Emmett–Teller (BET) methods. The surface area was calculated using the liner fitting of the Brunauer–Emmett–Teller (BET) equation with the range of *P*/*P*_0_ = 0.05–0.3. Surface morphology and EDX analysis of the samples were obtained using FEI Quanta 250 FEG microscope operating at 15 kV. The FTIR spectra of the samples were collected using a Bruker Vertex 7.0 Fourier transform infrared (FTIR) spectrometer with a scanning wavenumber (ranging from 400 to 4000 cm^−1^) and a spectral resolution of 2 cm^−1^. X-ray photoelectron spectrometer (XPS) spectra of fresh and spent catalysts were recorded on a Kratos AXIS Supra spectrometer with a monochromatic Al Kα X-ray (1486.6 eV) source for excitation. The binding energy of the chemical species was calibrated using the C 1s peak at 284.8 eV as an internal standard. The percentages of the individual elements were fitted by analysing the areas of the respective peaks (CasaXPS).

### 2.6 H-transfer reduction reaction

The H-transfer reduction reaction of catalysts with various aldehyde substrates were performed under batch conditions: 1.4 mmol aldehyde was dissolved in 2-propanol (4.6 mL) in a round bottom flask equipped with reflux condenser. Then 0.14 mmol 1,2,4,5-tetramethylbenzene were added as the internal standard. Then the catalyst was then added (∼0.4 mmol Al) and the reaction mixture was stirred at 90 °C. After reaction, 200 μL of the crude solution was extracted when after the reaction mixture cooling down to room temperature and then diluted with chloroform-d (CDCl_3_) for NMR analysis. The ^1^H NMR analyses was performed using a Bruker AVIII HD 400 MH_Z_ spectrometer, and the chemical shifts in ^1^H spectra were referenced to trimethylsilane (TMS). The conversion, selectivity and turnover frequency (TOF) was determined using the following equations:1
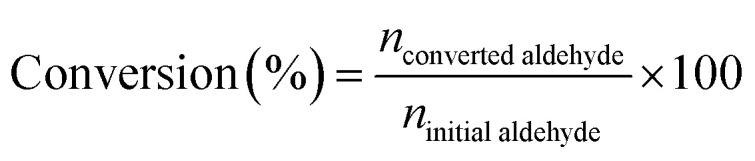
2
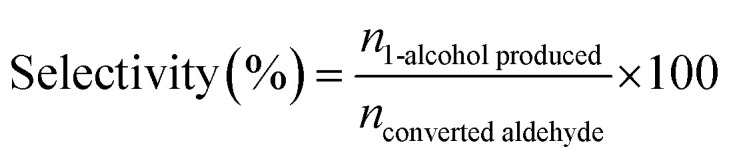
3
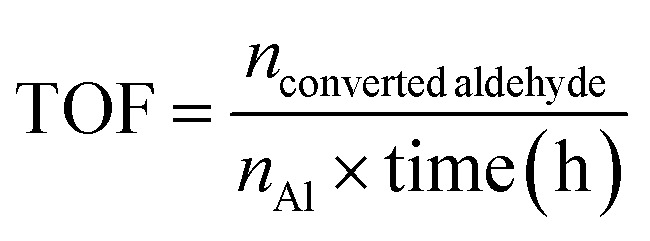


### 2.7 Catalysts recyclability tests

The catalyst recyclability tests were conducted with the modified Al–OTES–MgO and Al–DBB–MgO using hexanal reduction as the model reaction. The reaction condition was kept the same and after each reaction cycle, the spent catalysts were washed and dried for the subsequent cycles without any refresh treatment. After 5 reaction cycles, the catalysts were collected and washed for XPS and XRD analysis.

## 3. Results and discussions

### 3.1 Materials characterization

**Fig. 1:**
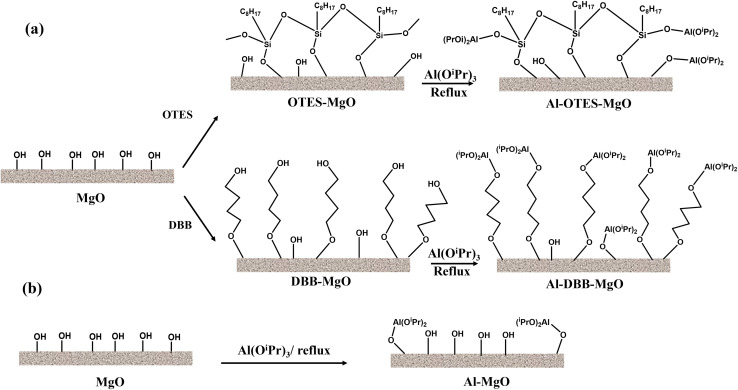
Processes of (a) MgO support modification followed by Al(O^*i*^Pr)_3_ immobilization (a); and (b) Al(O^*i*^Pr)_3_ immobilization onto MgO directly (b).

**Fig. 2:**
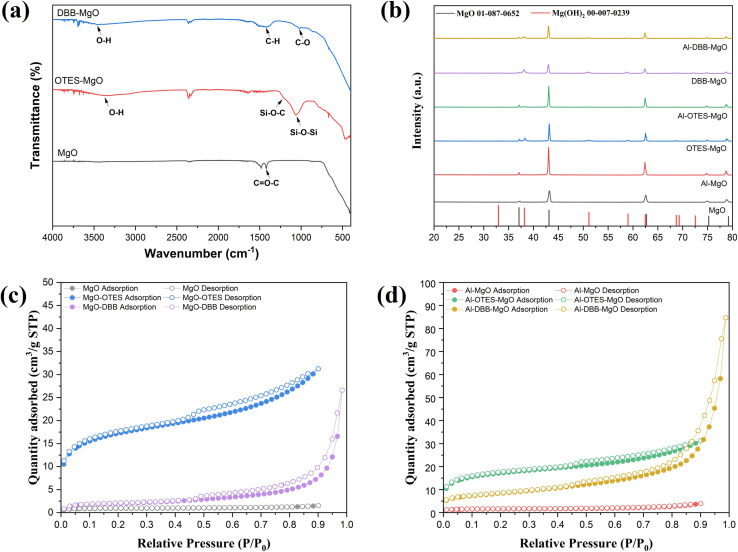
(a) FTIR spectra of MgO and modified MgO supports; (b) XRD patterns of supports and corresponding catalysts with JCPDS standard cards: 01-087-0652 (MgO) and 00-007-0239 [Mg(OH)_2_]; N_2_ adsorption–desorption isotherms of supports (c) and catalysts (d).

The modification of MgO support and the immobilization of Al(O^*i*^Pr)_3_ are illustrated in Fig. 1. The successful grafting of OTES onto MgO was confirmed by FTIR spectroscopy, as shown in Fig. 2a. For OTES–MgO, distinct peaks were observed at approximately 1080 cm^−1^, along with a smaller shoulder peak at 1260 cm^−1^, corresponding to the vibrational modes of Si–O–Si and Si–O–C bond, respectively.^17^ These peaks were absent in unmodified MgO. Furthermore, the grafted OTES formed densely packed self-assembled layers, which significantly increased the surface area from 2.9 m^2^ g^−1^ (for unmodified MgO) to 84.3 m^2^ g^−1^ for OTES–MgO (Table 1). This modification altered the surface properties of MgO, creating a “nest-like” structure, showing the characteristics of mesoporous materials. Evidence of this meso-porosity was provided by the N_2_ adsorption–desorption isotherm of OTES–MgO (Fig. 2c), which transformed from the non-porous nature of unmodified MgO (type II isotherm) to a type IV isotherms with moderate slop at higher *P*/*P*_0_ range (*P*/*P*_0_ > 0.8).^18^

**Table 1:** Textural properties and/or Al content of the supports and prepared catalysts

	Al (wt%)	*S*_BET_ (m^2^ g^−1^)
MgO	—	2.9
OTES–MgO	—	84.3
DBB–MgO	—	6.8
Al–MgO	0.25	5.1
Al–MgO–OTES	3.72	57.4
Al–DBB–MgO	11.42	29.5

Similarly, the addition of DBB to the MgO surface also increased the surface area, resulting a material with a surface area of 6.8 m^2^ g^−1^. The N_2_ adsorption–desorption isotherm of DBB–MgO (Fig. 2c and Table 1) exhibited a profile similar to that of bare MgO, with the steepest slope at higher relative pressures due to the high difference in volumes of gas adsorbed at the high-pressure points, indicating its microporous nature induced by the presence of the DBB carbon chain layer.^19,20^ This DBB layer acts as a “brush” on the MgO surface, consisting of extended hydrocarbon chains terminated with hydroxyl groups (–OH). The terminal bromine (Br) of DBB likely undergoes nucleophilic substitution with water (solvent) or surface hydroxyl groups on MgO, forming C–O bond.^21^ The FTIR spectrum of DBB–MgO (Fig. 2a) showed a characteristic C–O stretching peak at around 1050 cm^−1^.

Notably, the addition of OTES and DBB did not significantly alter the crystal structure of MgO, as indicated by the XRD patterns of both supports and catalysts (Fig. 2b). The diffraction peaks at 2*θ* values of 37.2°, 43.2°, 62.5°, 74.9° and 78.8° correspond to the (111), (200), (220), (311), and (222) planes of cubic polycrystalline MgO, consistent with the JCPDS No. 01-087-0652 standard.^22^ However, a minor Mg(OH)_2_ phase was detected in the modified MgO supports, as evidenced by additional diffraction peaks (marked with black circles) corresponding to the (101), (102), and (110) planes at 2*θ* = 38.2°, 51.0°, and 58.9° (JCPDS No. 00-007-0239), respectively.^23,24^ This phase likely resulted from surface hydrolysis induced by water.^25^ After immobilization, significant differences in crystal structure were not observed between the supports and the catalysts.

The immobilization of Al(O^*i*^Pr)_3_ was achieved through exchange reactions between the surface/terminated hydroxyl groups and the isopropyl linkers of Al(O^*i*^Pr)_3_.^3,26^ The amount of immobilized Al(O^*i*^Pr)_3_ was determined by measuring the Al loading *via* ICP-OES, with results summarized in Table 1. Among the samples, Al–MgO exhibited the lowest Al loading, which can be attributed to its minimal surface area. Furthermore, due to the relatively large size of the Al(O^*i*^Pr)_3_ molecule, its grafting onto the MgO surface likely hindered access to adjacent hydroxyl groups, thereby limiting further immobilization.

In contrast, the Al loading increased significantly after the MgO surface was modified with OTES or DBB, with Al–DBB–MgO showing the highest Al loading. Both OTES and DBB contain terminal hydroxyl groups that can facilitate the immobilization of Al(O^*i*^Pr)_3_. For Al–OTES–MgO, the Al(O^*i*^Pr)_3_ was likely grafted primarily onto the outer layer of OTES, as the densely packed OTES near the MgO surface hindered access to inner hydroxyl groups. Additionally, the lower surface area of MgO limited the amount of OTES grafting, reducing the number of hydroxyl groups available for further Al(O^*i*^Pr)_3_ immobilization. The grafting of Al(O^*i*^Pr)_3_ may have also occupied some of the cavity space within the OTES framework, contributing to a decrease in surface area.

Conversely, the DBB modification significantly increased the number of the terminal hydroxyl groups. The flexibility of carbon chains of DBB also minimized steric hindrance during Al(O^*i*^Pr)_3_ grafting, resulting in the highest Al loading among the samples. The densely grafted Al(O^*i*^Pr)_3_ molecules formed a onto DBB–MgO might pack a also contributed to the overall surface area, explaining the increased surface area observed for Al–DBB–MgO.

**Fig. 3:**
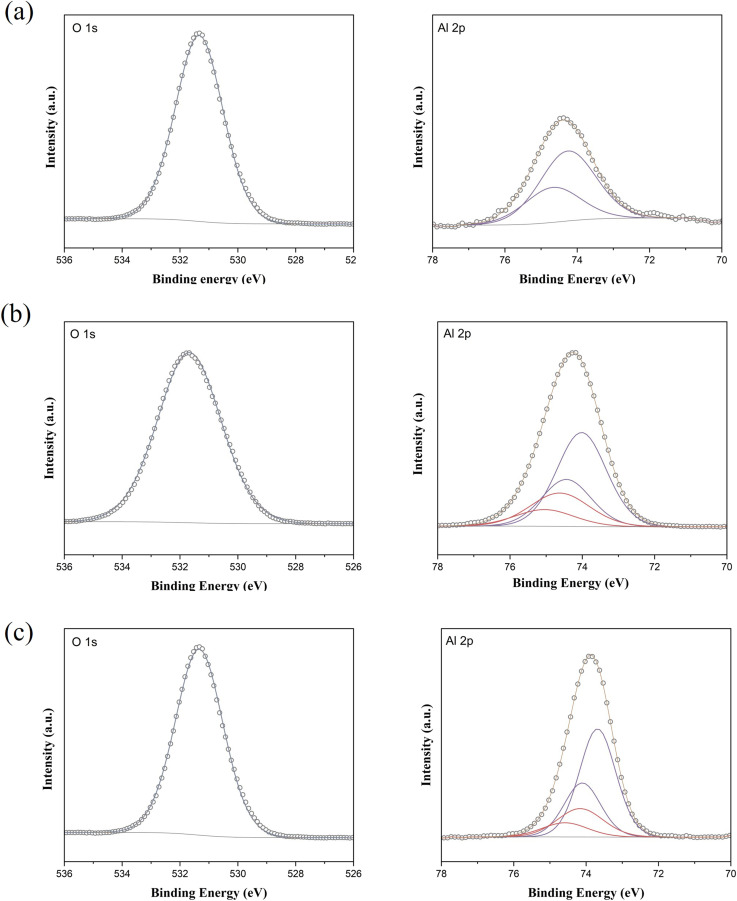
XPS spectra of O 1s and Al 2p in the fresh catalyst (a) Al–MgO; (b) Al–OTES–MgO and (c) Al–DBB–MgO.

The surface chemical states of the fresh catalysts were further examined using X-ray photoelectron spectroscopy (XPS), with the Al 2p and O 1s spectra presented in Fig. 3. The O 1s peak in all samples appeared as a single peak at approximately 531.7 eV, corresponding to hydroxyl groups on the MgO surface or terminated hydroxyl groups introduced by the OTES and DBB functional groups. Due to the similar binding energies of these two types of hydroxyl groups, distinguishing them from one another is challenging. The Al 2p peak confirmed the successful immobilization of Al(O^*i*^Pr)_3_ molecules onto the support surfacers. The Al–MgO exhibited a single Al 2p doublet peak, indicative of Al–O bond formation between Al(O^*i*^Pr)_3_ and the surface hydroxyl groups. In contrast, the modified catalyst, Al–OTES–MgO and Al–DBB–MgO, displayed two doublet Al 2p peaks, suggesting that Al(O^*i*^Pr)_3_ molecules were anchored both on the MgO surface (at 74.2–74.6 eV) and on the terminated hydroxyls groups from the OTES or DBB modifiers (at 73.7–74.0 eV), with the area ratio of around 0.25 (80% terminated –OH to 20% surface –OH). These findings support our hypothesis that hydroxyl groups introduced by the modification process serve as active sites for catalyst grafting and play a crucial role in facilitating the subsequent hydrogen transfer reactions.^27,28^

**Fig. 4:**
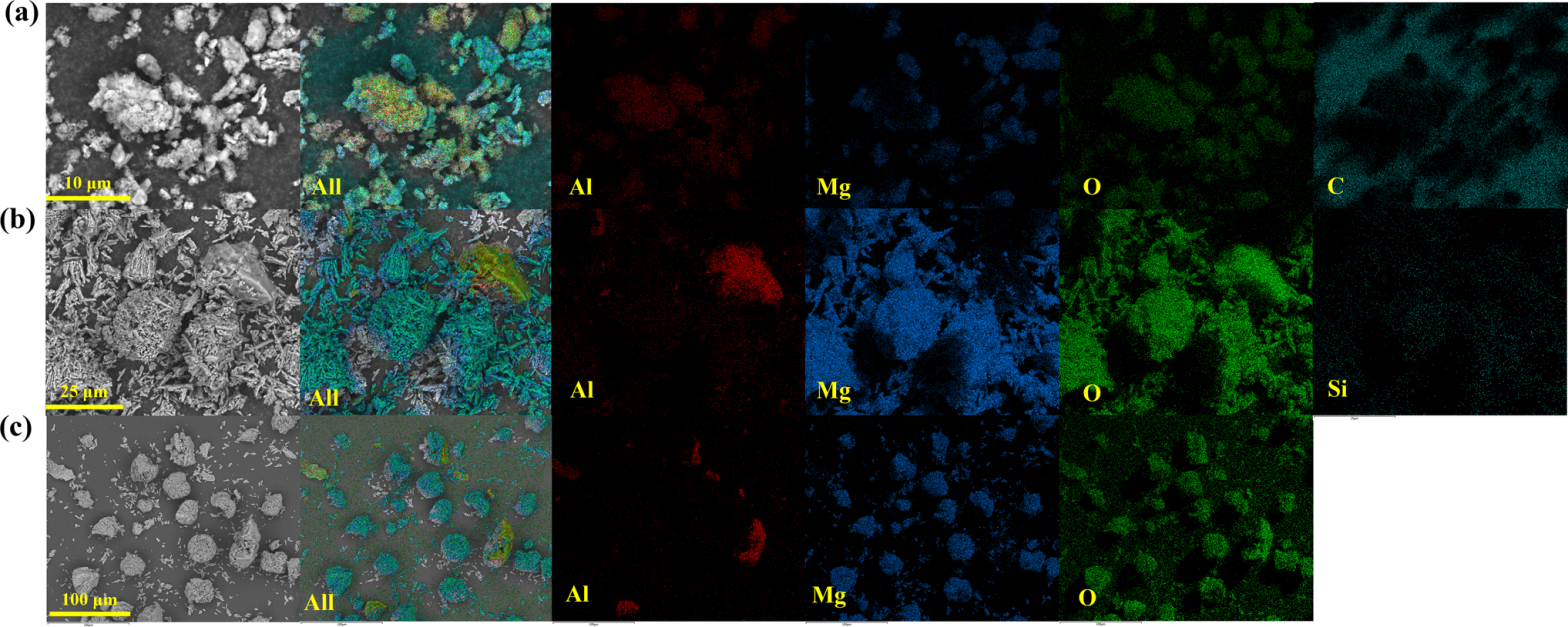
SEM images and EDX elemental maps of catalysts: (a) Al–MgO; (b) Al–OTES–MgO; (c) Al–DBB–MgO. Elemental maps of Al (red), Mg (blue), O (green) and Si or C (turquoise blue) are shown.

The morphology and distribution of relevant elements were further analysed using SEM and EDX mapping, as shown in Fig. 4. The SEM images revealed an intensive and uniform distribution of carbon on DBB–MgO, whereas only a small amount of silicon was detected on OTES–MgO. This supports the conclusion that limited OTES was grafted onto the MgO surface. As SEM primarily examines surface features, the detected silicon distribution likely originated from the outer layer of the OTES coating. All catalyst samples showed the presence of aluminum, confirming the successful immobilization of Al(O^*i*^Pr)_3_.

### 3.2 H-transfer reaction activity of the heterogenized catalysts

The catalytic activity of the materials for the H-transfer reaction was evaluated using a range of substrates, including saturated and unsaturated aldehydes and ketones, with 2-proponal serving as both the solvent and hydrogen donor. The activity data are summarised in Table 2.

**Table 2:** H-transfer reduction activities of aldehydes and ketones over modified and unmodified Al–MgO catalysts

Substrate	Structure	Catalyst	Conversion (%)	Selectivity (%)	TOF^a^ (h^−1^)
Hexanal	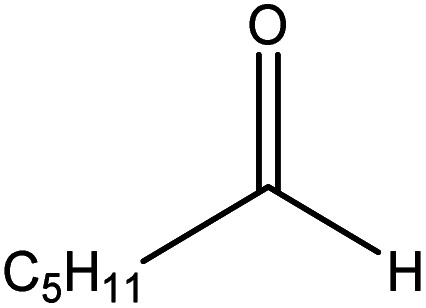	Al–DBB–MgO	46.6	92.7	0.21
		Al–OTES–MgO	22.9	87.2	0.14
		Al–MgO	12.8	>99	0.06
Cyclohexanone	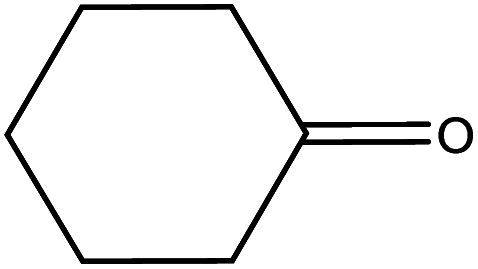	Al–DBB–MgO	28.1	>99	0.13
		Al–OTES–MgO	22.3	92.1	0.10
		Al–MgO	5.6	>99	0.03
Benzaldehyde	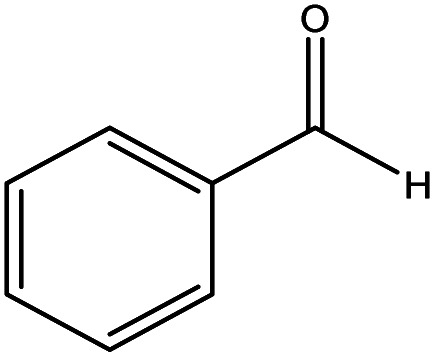	Al–DBB–MgO	23.9	84.4	0.23
		Al–OTES–MgO	13.0	87.5	0.12
		Al–MgO	7.8	94.6	0.07
Cinnamaldehyde	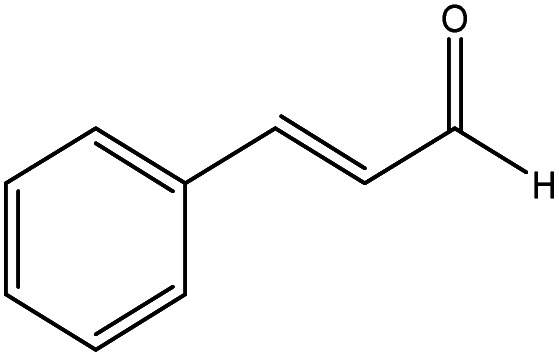	Al–DBB–MgO	12.6	96.5	0.12
		Al–OTES–MgO	6.7	>99	0.05
		Al–MgO	4.6	>99	0.02

a TOF was calculated as the average value (over 3 h).

As previously reported,^29^ the reaction proceeds *via* a cyclic six-membered transition state involving direct hydrogen transfer from the H-donor (alcohol) to the H-acceptor (aldehyde or ketone). Among the substrates tested, saturated aldehydes were generally easier to reduce than ketones, particularly liner chain aldehydes. For example, all heterogenized catalysts demonstrated the highest activity in reducing hexanal compared to cyclohexanone. The reduced activity observed for cyclohexanone is likely due to the steric hindrance, which restricts its coordination of the ketones to the Al centre.^30^ In the case of unsaturated aldehydes, such as cinnamaldehyde, a typical α,β-unsaturated aldehyde with a conjugated aromatic ring and C=C bond, exhibited the poorest conversion. This reduced activity can be attributed to the combined influence of steric and electronic effects, resulting in the lowest observed activity.

Catalyst based on the unmodified MgO demonstrated the lowest activity among various substrates, owning to the lowest Al(O^*i*^Pr)_3_ grafting. In contrast, the modified catalysts demonstrated significantly improved activity across all the substrates, achieving approximately 2- to 4-fold increase compared to unmodified catalyst. Moreover, the heterogeneous catalysis followed the same substrate-specific trends as the homogeneous process, suggesting a similar reaction mechanism for the classical H-transfer reduction.

When normalised by turnover frequency (TOF, last entry in Table 2), Al–DBB–MgO outperformed the other solid catalysts. This superior performance can be attributed to the flexibility of the DBB layer grafted onto MgO, where rotation of the carbon chains reduces steric limitations, facilitating coordination between the Al canter and the substrates. Additionally, the structural similarity between DBB and certain substrates, such as hexanal, enhances reactant adsorption through stronger interaction.

For Al–OTES–MgO, an activity increase was also observed compared to the unmodified catalyst. The enhanced surface area of Al–OTES–MgO is thought to improve contact between the reactants and active sites. However, the densely packed OTES layer induces significant steric hindrance, which was particularly detrimental for substrates with ring structures. This may explain the comparatively lower activity of catalyst Al–OTES–MgO for such substrates.

**Fig. 5:**
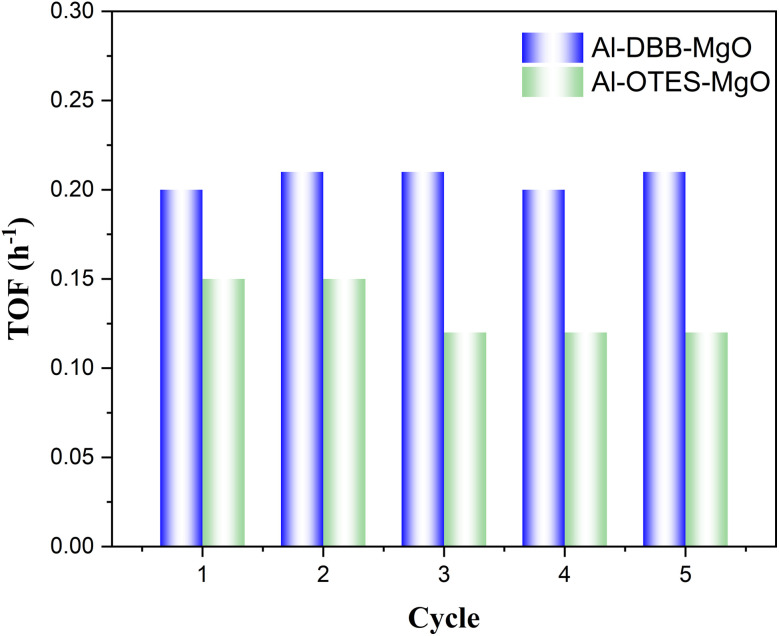
Recycling performance of modified Al–MgO catalysts (Al–DBB–MgO and Al–OTES–MgO) for the H-transfer reduction of hexanal.

To further evaluate the impact of the support modification on catalytic performance, we conducted recyclability tests on the modified catalysts Al–DBB–MgO and Al–OTES–MgO using hexanal reduction as a model reaction. As shown in Fig. 5, the turnover frequency (TOF) of Al–DBB–MgO remained stable after 5 reaction cycles, indicating excellent catalytic stability. While the TOF of Al–OTES–MgO dropped by around 14%, likely due to the leaching of Al(O^*i*^Pr)_3_.

**Fig. 6:**
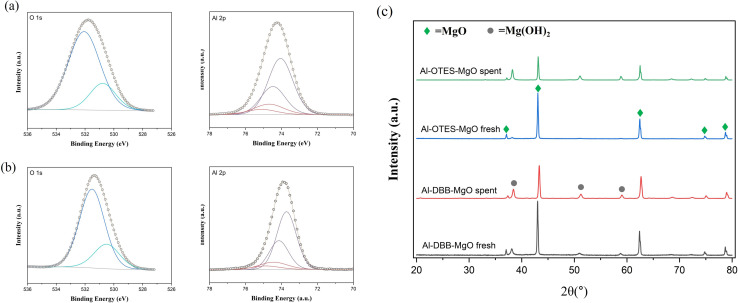
XPS analysis of spent Al–OTES–MgO (a) and Al–DBB–MgO (b); XRD patterns of fresh and spent catalyst for comparison (c).

XPS and XRD analyse were performed on the spent catalysts after the recyclability tests investigate potential structural and surface property changes during the reaction. As depicted in Fig. 6a and b, an additional peak emerged at approximately 530.5 eV with an area of ∼20%, suggesting the formation of the more reduced oxygen species, which can be attributed to Mg(OH)_2_. The XRD patterns of the spent catalysts, as shown in Fig. 6c, further supported these fundings, showing an increased intensity of the diffraction peaks at 2*θ* = 38.2°, 51.0°, and 58.9°, corresponding to the (101), (102), and (110) planes of Mg(OH)_2_. This increment is likely due to accumulated surface hydrolysis accelerated by water during the catalyst recovery and washing. Additionally, oxidation state of Al remained largely unchanged. These observations confirm that the modified catalysts retain their structural integrity upon recycling, with only limited surface changes.

## 4. Conclusion

This study investigated the effect of surface modification of MgO supports and subsequent immobilization of Al(O^*i*^Pr)_3_ to produce catalytic materials H-transfer reductions. The surface modifications with OTES and DBB significantly altered the surface properties of MgO, as evidenced by the increased surface areas, engineered porosity, and surface functionalization with terminal hydroxyl groups, providing active sites for efficient Al(O^*i*^Pr)_3_ grafting.

Among all the solid catalysts tested, Al–DBB–MgO demonstrated the highest catalytic activity, which can be attributed to the flexibility of the DBB carbon chains, facilitating enhanced reactant coordination. In comparison, Al–OTES–MgO showed moderate improvements in activity; however, the steric hindrance from densely packed OTES layers limited its performance, particularly with bulky substrates. Additionally, both Al–DBB–MgO and Al–OTES–MgO demonstrated remarkable stability during the cycling tests with minor structure change. These findings highlight the critical role of optimizing the surface chemistry of support materials to enhance catalytic performance.

## Data availability

The data that support the findings of this study are available within the article.

## Author contributions

Xiao Yu: conceptualisation, investigation, formal analysis, visualization, writing – original draft; Atika Muhammad: investigation, visualization, writing – original draft; Boya Qiu: methodology, validation, writing; Aristarchos Mavridis: methodology, investigation; Min Hu: supervision, writing – review & editing; Carmine D'Agostino: conceptualization, supervision, resources, funding acquisition, project administration, writing – review & editing.

## Conflicts of interest

There are no conflicts to declare.
